# Microstructural Evolution and Dynamic Softening Mechanisms of Al-Zn-Mg-Cu Alloy during Hot Compressive Deformation

**DOI:** 10.3390/ma7010244

**Published:** 2014-01-08

**Authors:** Cangji Shi, Jing Lai, X.-Grant Chen

**Affiliations:** Department of Applied Science, University of Québec at Chicoutimi, Saguenay, Québec G7H 2B1, Canada; E-Mails: cang-ji.shi@uqac.ca (C.S.); jing.lai@uqac.ca; (J.L.)

**Keywords:** Al-Zn-Mg-Cu alloy, hot compression, microstructure, decline ratio map, flow stress, dynamic softening mechanism

## Abstract

The hot deformation behavior and microstructural evolution of an Al-Zn-Mg-Cu (7150) alloy was studied during hot compression at various temperatures (300 to 450 °C) and strain rates (0.001 to 10 s^−1^). A decline ratio map of flow stresses was proposed and divided into five deformation domains, in which the flow stress behavior was correlated with different microstructures and dynamic softening mechanisms. The results reveal that the dynamic recovery is the sole softening mechanism at temperatures of 300 to 400 °C with various strain rates and at temperatures of 400 to 450 °C with strain rates between 1 and 10 s^−1^. The level of dynamic recovery increases with increasing temperature and with decreasing strain rate. At the high deformation temperature of 450 °C with strain rates of 0.001 to 0.1 s^−1^, a partially recrystallized microstructure was observed, and the dynamic recrystallization (DRX) provided an alternative softening mechanism. Two kinds of DRX might operate at the high temperature, in which discontinuous dynamic recrystallization was involved at higher strain rates and continuous dynamic recrystallization was implied at lower strain rates.

## 1. Introduction

The 7xxx series aluminum alloys are very attractive materials to be used in the automotive and aerospace industries, due to their excellent combination of properties, such as high strength-to-density ratio, high fracture toughness and resistance to stress corrosion cracking [1]. A good understanding of the hot deformation behavior and microstructural evolution is of primary importance for the design of hot-forming processes, such as rolling, extrusion and forging. Thermomechanical factors, such as the degree of deformation, deformation temperature and strain rate, are the main factors that influence the flow stress and the associated microstructure [2,3]. Furthermore, the evolution of flow stress is correlated with different dynamic softening mechanisms during hot deformation at various deformation conditions [2,4].

Dynamic recovery (DRV) and dynamic recrystallization (DRX) are the typical softening mechanisms in metals and alloys during deformation at elevated temperatures [2,4]. Aluminum and its alloys with high stacking fault energy exhibit a high rate of DRV, which significantly inhibits DRX [2]. However, the formation of new grains during hot deformation of aluminum alloys was frequently reported, while several mechanisms of DRX for aluminum alloys have been proposed [5–13]. The discontinuous dynamic recrystallization (DDRX) was observed in aluminum and aluminum alloys by many researchers [5–9]. The DDRX involves the development of high angle grain boundaries via nucleation and growth of new grains, which typically initiates at high angle boundaries, such as original grain boundaries, boundaries of dynamically recrystallized grains and boundaries created during deformation [4]. The bulging of grain boundaries is frequently observed as a prelude to DDRX, and subsequent grain growth is processed by strain-induced boundary migration [4]. Furthermore, continuous dynamic recrystallization (CDRX) was also observed in aluminum alloys [10–13]. This mechanism differs from that of DDRX, in which new grains are formed progressively within the deformed original grains from a continuous increase of subgrain boundary misorientations, as a result of the accumulation of dislocations in low angle boundaries [2,10–13].

Moreover, during the plastic deformation process, most of the deformation work is converted to heat [14]. Plastic deformation performed at high strain rate conditions is essentially adiabatic, thus generating a significant deformation heat and leading to a noticeable increase in temperature [15]. The deformation heating may cause the thermal softening of materials, which offsets the work hardening effect and result in a decrease in flow stress [16–18]. In addition, the formation of adiabatic shear bands is usually observed at regions where deformation is localized [15]. Furthermore, precipitation hardening alloys may alter dynamic softening behavior as a result of changes in precipitate morphology, when the material is not thoroughly over-aged. Materials pre-aged at deformation temperature have a low peak stress and exhibit a gradual softening as precipitates coalesce and subgrains form [19,20]. Solution-treated aluminum alloys exhibit a high peak stress due to dynamic precipitation (DPN), followed by a rapid dynamic softening as precipitates coalesce, solute depletes and DRV progresses [17,21–24].

To date, the previous studies have been mainly on determining the Zener–Hollomon parameter (Z), the activation energy for hot deformation and various material constants in the constitutive equations for describing the hot deformation behavior of 7xxx aluminum alloys [25,26]. The softening mechanism of these alloys is generally reported to be DRV at low temperature with a high strain rate, while DRX occurs as deformation at a high temperature with a low strain rate [27–29]. Different dynamic recrystallization mechanisms of 7075-T6 aluminum alloy have been studied during deformation at a semi-solid temperature range (450–580 °C) [30]. Several researchers have studied the kinetics and rate of DRX and DRV using various models [31,32]. Jonas *et al.* [31] measured the kinetics of DRX using the Avrami formulation and predicted the DRX flow curves. Mostafaei and Kazeminezhad [32] proposed a mathematical method based on hot flow curves to predict the effects of temperature and strain rate on the kinetics of DRV in the Al-Mg alloy. However, quantitative characterization of the evolution of flow stress curves at different deformation conditions has rarely been reported. The relationship between the evolution of flow stress and various dynamic softening mechanisms during hot deformation needs to be clarified. During deformation at a medium temperature range (up to 450 °C), a systematical investigation of different dynamic softening mechanisms at various deformation conditions for 7150 aluminum alloy is limited.

In the present paper, the hot deformation behavior of an Al-Zn-Mg-Cu alloy (7150) is studied using uniaxial compression tests performed at various temperatures and strain rates. The microstructural evolution during the hot deformation is investigated in order to understand the various dynamic softening mechanisms at different deformation conditions. Based on the different decline levels in flow stresses with respect to peak stresses, a decline ratio map of flow stresses is proposed to correlate flow stress behavior with dynamic softening mechanisms at various deformation conditions.

## 2. Experimental

The experiments were conducted on an Al-Zn-Mg-Cu alloy. The chemical composition of the experimental alloy analyzed by an optical emission spectrometer is given in Table 1. Most of the elements of the alloy are in the range of 7150 composition designation. However, no Zr is added in this study. Approximately 3 kg of materials were melted in an electrical resistance furnace and then cast into a rectangular permanent steel mold measuring 30 × 40 × 80 mm^3^. The cast ingots were homogenized at 465 °C for 24 h, followed by a direct water quenching. Cylindrical samples 10 mm in diameter and 15 mm long were machined from the homogenized ingots. Uniaxial compression tests were conducted on a Gleeble 3800 thermomechanical simulation unit at strain rates of 0.001, 0.01, 0.1, 1 and 10 s^−1^ and deformation temperatures of 300, 350, 400 and 450 °C, respectively. During the tests on the Gleeble unit, the samples were heated to the desirable deformation temperature at a heating rate of 10 °C/s and held for 3 min to ensure a homogenous temperature distribution through the samples. The samples were deformed to a total true strain of 0.8 and then immediately water-quenched to maintain the microstructure at the deformation temperature. Some selected samples were also deformed to different true strains of 0.1, 0.3 and 0.5, followed by water quenching, in order to investigate the microstructural evolution during the deformation process.

The microstructures of the as-cast and as-homogenized materials were examined prior to hot deformation under an optical microscope. The intermetallic phases were identified using the electron backscattered diffraction (EBSD) technique and the energy dispersive spectroscope (EDS) with a scanning electron microscope (SEM, JEOL JSM-6480LV, JEOL Ltd., Tokyo, Japan). All deformed samples were sectioned parallel to the compression axis along the centerline and then polished and etched in Keller solution for optical microscope observation. Additionally, some deformed and as-homogenized samples were selected for EBSD analysis. In the EBSD analysis, the boundaries of both the grains and subgrains are defined as low angle boundaries (LABs), medium angle boundaries (MABs) and high angle boundaries (HABs) for which the misorientation angles of boundaries occur in the ranges of 1°−5°, 5°−15° and greater than 15°, respectively [33]. The step size between the scanning points was set to 1.0 μm for the grain structure of the samples. The dynamic recrystallized grains were separated from the deformed ones using the grain average misorientation (GAM) method [34,35]. A threshold value of GAM (2.5°) inside a grain was determined to distinguish a recrystallized grain from a deformed one. Grains with GAM less than 2.5° were considered to be recrystallized grains. Subsequently, the size of the recrystallized grains was measured by means of a grain reconstruction method [36]. For quantitative measurement of the misorientation distribution of boundaries, am EBSD line scan was carried out [36,37], and a sample surface area of about 1.2 mm^2^ with a scanning step size of 3.0 μm was selected. In addition, EBSD analysis was performed to measure the grain size of the as-homogenized sample using the linear intercept method [36]; a surface area of approximately 10 mm^2^ with a scanning step size of 5.0 μm was selected for the sample. Samples for TEM observation were mechanically ground to the thicknesses of 35–60 μm followed by electropolish in a twin-jet polishing unit, which was operated at 15 V and −20 °C using a 30% nitric acid and 70% methanol solution. The disks were observed in a transmission electron microscope (TEM, JEOL-JEM-2100), operated at 200 kV.

## 3. Results

### 3.1. Initial Microstructure

**Figure 1.**
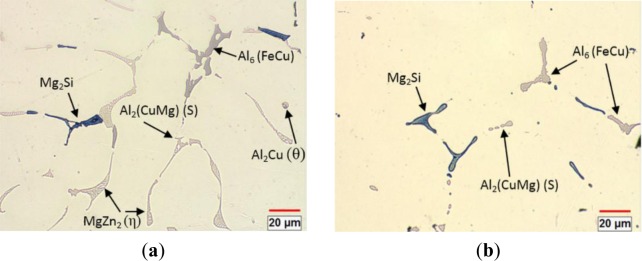
The optical micrographs of the alloy: (**a**) as-cast microstructure; and (**b**) as-homogenized microstructure.

Figure 1a shows the initial as-cast microstructure of the alloy, which contained MgZn_2_(η), Al_6_(FeCu), Mg_2_Si, Al_2_CuMg(S) and Al_2_Cu(θ) intermetallic phases, distributed in the interdendritic boundaries. The Al_2_CuMg phase was observed to be attached with the MgZn_2_ phase (lamellar eutectic morphology) and Al_2_Cu phase (isolated droplet morphology) in the aluminum matrix. These phases have been commonly observed in as-cast 7xxx aluminum alloys [38,39]. After homogenization treatment at 465 °C for 24 h, the MgZn_2_ and Al_2_Cu phases were completely dissolved into the aluminum matrix, while the coarse particles of Al_6_(FeCu), Mg_2_Si and Al_2_CuMg were only dissolved partially, and some of them were retained in the matrix (Figure 1b).

### 3.2. Flow Stress Behavior

**Figure 2.**
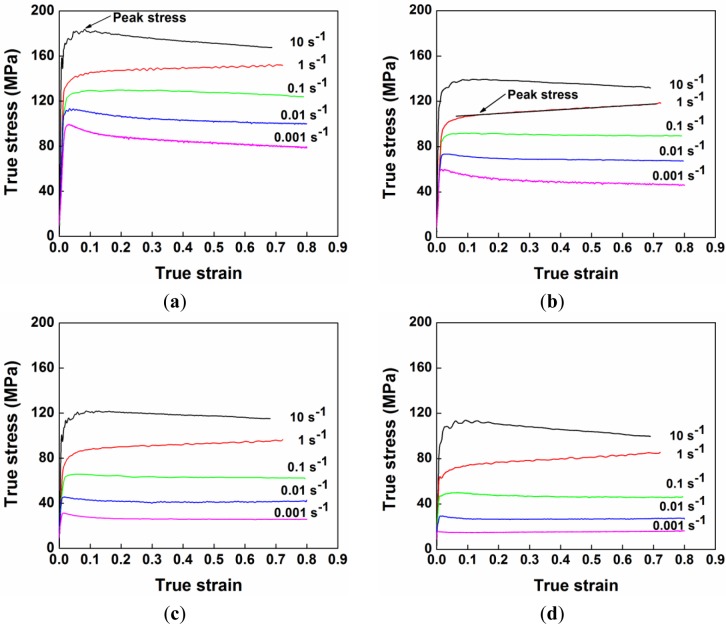
True stress-true strain curves of the alloy during hot compression deformation: (**a**) *T* = 300 °C; (**b**) *T* = 350 °C; (**c**) *T* = 400 °C; and (**d**) *T* = 450 °C.

The hot compression tests of the alloy were carried out at deformation temperatures of 300 to 450 °C and at strain rates of 0.001 to 10 s^−1^. A series of true stress-true strain curves under different deformation conditions are shown in Figure 2. In general, the flow stress increased rapidly at the beginning of deformation and then remained fairly constant or decreased to some extent after attaining the peak stress. At the early stage of deformation, dislocations multiplied dramatically, and the work hardening process was predominant, thereby leading to a rapid increase in the flow stress [4]. As the dislocation density increased, dynamic softening occurred, which can offset the effect of work hardening [2,4]. Thus, the flow stress increased at a decreasing rate until the peak stress was reached. Subsequently, the flow stress either decreased with increasing strain or remained fairly steady. The former behavior is observed when the rate of dynamic softening is higher than that of work hardening [2,4]. The latter behavior occurs as a result of a dynamic equilibrium between work hardening and dynamic softening [2,4]. In both cases, a distinct peak stress is visible as the maximum value in the flow stress curve (see the arrow in Figure 2a). Occasionally, the flow stress can continuously increase after the early stage of deformation. In this case, the peak stress was identified as the tangent point on the flow stress curve by the extension of a line along the steady-state flow stresses (see the arrow in Figure 2b).

Furthermore, it is evident that the level of the flow stress decreased with increasing deformation temperature and with decreasing strain rate. As the deformation temperature rises, the thermal activation, which favors overcoming an energy barrier to dislocation motion, is increased, while the stress needed to deform a material decreases [40,41]. Besides, with the increase of temperature, the level of dynamic softening is improved, so that the dislocation density is reduced to facilitate the further dislocation motion [2,4]. Hence, increasing temperature could substantially reduce the resistance to dislocation movement, which results in the decline of flow stress. On the other hand, with the decrease of the strain rate, the dislocation multiplication rate is reduced, which leads to less tangled dislocation structures as barriers to the dislocation movement [2]. In addition, a decreasing strain rate results in an increasing level of dynamic softening, due to the relatively more time for the proceeding of dislocation polygonization, which facilitates the further movement of dislocation [2,4]. Therefore, decreasing the strain rate is expected to lower the stress, which is needed for dislocation movement, and leads to the decrease in flow stress.

### 3.3. Decline Ratio Map of Flow Stresses

**Figure 3.**
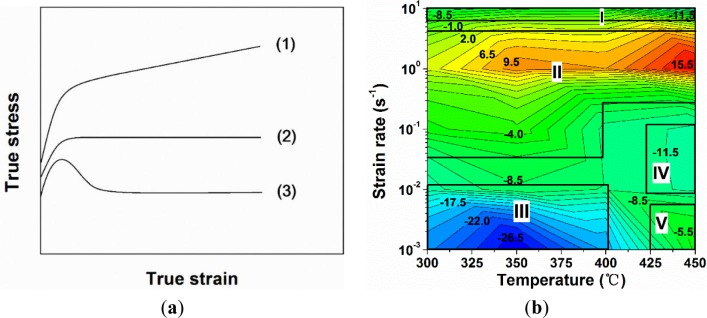
(**a**) Schematic illustration of the evolution of true stress-true strain curves in three cases; (**b**) decline ratio map of flow stresses, *R_d_* (%), as a function of deformation temperature and strain rate.

The flow stress curves can be divided in three typical cases, as shown in Figure 3a, which are closely related to different dynamic softening mechanisms. In the first case, the flow stress rises rapidly and then undergoes a continuous increase of stress with increasing strain. A progressive increase of flow stress is likely due to the rate of work hardening being higher than that of DRV [4]. In the second case, the flow stress increases to a plateau, followed by a fairly steady stress. This indicates that a dynamic equilibrium between work hardening and DRV is achieved [4]. In the third case, the flow stress exhibits a distinct peak value and then decreases significantly. In general, the decline of flow stress can be associated with the coalescence of precipitates, enhanced DRV [17,21–24] and DDRX [5–9]. Under conditions of low a Zener–Hollomon parameter, multiple peaks may be exhibited, along with a continuous flow softening, which is also a result of DDRX [4]. Cracking may also occur during hot deformation at high strain rates that could result in a decrease in the flow stress after reaching the peak stress [42,43]. In addition, the flow stress decrease can be caused by severe deformation heating when the deformation is performed at high strain rates [2,16–18].

A decline ratio of flow stress, *R_d_* is introduced to characterize the evolution of flow stress curves during the hot deformation of the alloy. *R_d_* is based on the decline level in flow stress at the end of the deformation with respect to the corresponding peak stress and is defined in Equation (1). The lower the value of *R_d_*, the more decline the flow stress relative to the peak stress.

$$R_{d}(\%)=\frac{{\text{σ}}_{s}-{\text{σ}}_{p}}{{\text{σ}}_{p}}\times 100$$ (1)

where σ*_s_* is the value of flow stress at the end of the deformation (at a true strain of 0.8 in our study) and σ*_p_* is the value of peak stress.

According to the experimental data obtained under deformation at temperatures of 300 to 450 °C and at strain rates of 0.001 to 10 s^−1^, the decline ratio map of flow stresses at various deformation conditions for the 7150 alloy is presented in Figure 3b. Generally, with increasing strain rate, the value of the decline ratio is increased, indicating an enhanced work hardening effect and a decreasing level of dynamic softening. However, at the strain rate of 10 s^−1^, the decline ratio value again decreased. This map can be divided into five deformation domains:

- Domain I illustrates a significant decrease in flow stress after the peak stress with the values of *R_d_* between −13% and −6%, when the deformation was performed at a high strain rate of 10 s^−1^.
- Domain II represents the deformation conditions at temperatures of 300 to 400 °C with strain rates of 0.1 to 1 s^−1^, as well as at higher temperatures of 400 to 450 °C with a strain rate of 1 s^−1^. Either a slight decrease or a continuous increase in flow stress after the peak stress is observed, and the values of *R_d_* vary between −8% and 17%.
- Domain III represents the deformation performed at temperatures of 300 to 400 °C with low strain rates of 0.001 to 0.01 s^−1^. A significant decline in flow stress is observed with the values of *R_d_* between −28% and −10%.
- Domain IV is characterized by a large decrease of flow stress with the value of *R_d_* approximately −12%, during the deformation at a high temperature of 450 °C with strain rates of 0.01 to 0.1 s^−1^. It is of interest to notice that only a single peak following a steady-state flow was observed in the flow stress curves of Domains III and IV (Figure 2). However, Yamagata [5,6] has reported that multiple peaks followed by a continuous flow softening could also be induced as a result of DDRX in pure aluminum at those deformation conditions. In both cases, the values of *R_d_* could be in the same range, though the softening mechanisms resulting in such features might be different.
- Domain V shows a slight decrease of flow stress under the deformation at a high temperature of 450 °C with a low strain rate of 0.001 s^−1^, and the value of *R_d_* is around −5%.

It is observed that *R_d_* is generally a function of temperature and strain rate, rather than a unique value. The changes of the values of *R_d_* in the Al-Zn-Mg-Cu alloy under different deformation domains correspond to various softening mechanisms [2,4–9,17,21–24,42,43], which will be discussed in the following sections.

### 3.4. Microstructural Evolution

**Figure 4.**
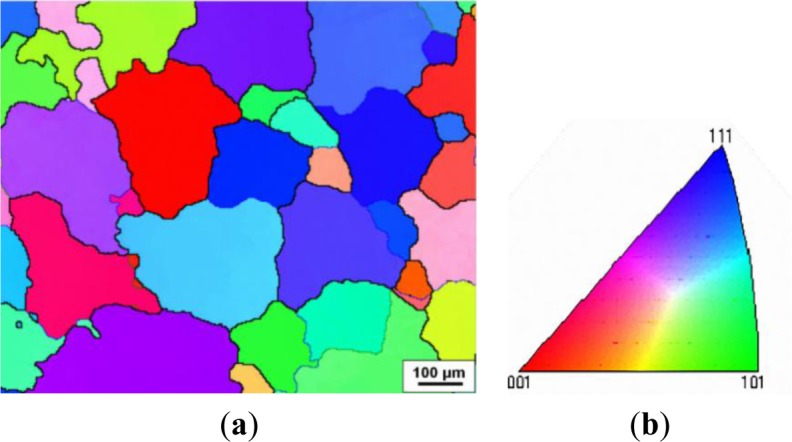
(**a**) Grain structure of the alloy after homogenization treatment; (**b**) representation of the color code used to identify the crystallographic orientations on a standard stereographic projection (red: [001]; blue: [111]; green: [101]).

The microstructures of deformed samples in different deformation domains were examined using the optical microscope. Besides, in an attempt to gain more insight into microstructural evolution during hot deformation, orientation imaging maps were generated at the same deformation conditions as those in optical micrographs. In an orientation imaging map, the boundary misorientation angles of both grains and subgrains can be distinguished by the colors: white lines: 1°−5°; blue lines: 5°−15°; thin black lines: 15°−30°; and thick black lines: (>30°). Figure 4 illustrates the initial grain structure of the homogenized sample, which is composed of uniform equiaxed grains that originate from the casting. The average grain size was 127 μm, and the grain boundaries are characterized by high angle boundaries, typically with misorientation angles between 30° and 60°.

**Figure 5.**
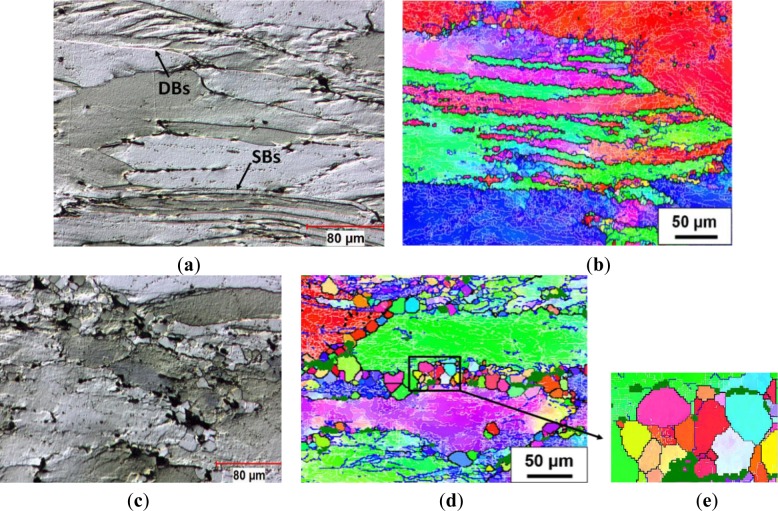
Optical micrographs and orientation imaging maps showing deformed microstructures under different deformation conditions: (**a**,**b**) 300 °C, 10 s^−1^; and (**c**–**e**) 450 °C, 10 s^−1^.

During hot compression, the original grains were plastically elongated perpendicular to the compression direction (Figure 5). In Domain I, when the alloy was deformed at 300 °C and 10 s^−1^, the original grains were severely torn and broke into irregular deformation bands (DBs; see the arrow in Figure 5a), due to deformation occurring on different slip systems [33]. Shear bands (SBs, see the arrow) were also observed through several grains, as a result of a highly localized plastic deformation. Figure 5b shows several deformation bands with high angle transition boundaries inside the elongated grains, which oriented along the elongation direction. Moreover, a large amount of low-angle boundaries were observed with misorientation angles largely between 1° and 5°, indicating a high density of cell and subgrain structures.

As the deformation temperature increased to 450 °C with a strain rate of 10 s^−1^, Figure 5c,d reveals that, besides the major recovered structure, strings of equiaxed grains with high angle boundaries (>15°) have been developed along the original grain boundaries and in regions that were associated with the large intermetallic particles (>1 μm). The dark green regions in Figure 5d,e represent the intermetallic particles, where no indexing of the EBSD pattern of aluminum occurs. This indicates that the formation of those recrystallized grains was closely related to the particle stimulated nucleation by large intermetallic particles [2,4,12]. Furthermore, the newly formed grains are distinguished by being free of an internal substructure (cell and subgrain structures). This suggests that they were formed as a result of static recrystallization during the quenching process. This phenomenon was also observed in AA1100 and AA5083 aluminum alloys during quenching after deformation at high temperatures and high strain rates due to considerable stored energy for static recrystallization [44,45].

**Figure 6.**
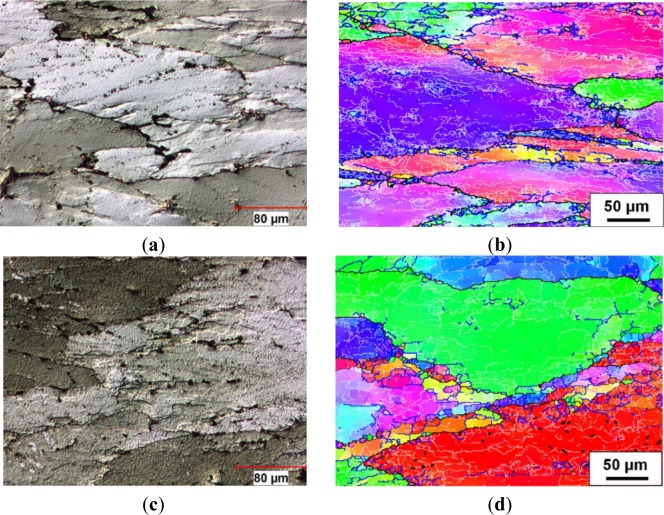
Optical micrographs and orientation imaging maps showing deformed microstructures under different deformation conditions: (**a**,**b**) 350 °C, 0.1 s^−1^; and (**c**,**d**) 350 °C, 0.001 s^−1^.

In Domain II, under the deformation condition at 350 °C and 0.1 s^−1^, Figure 6a shows that the deformation became more homogeneous compared to that in Figure 5a, with fewer deformation bands visible under the optical microscope. This is the result of an increase in the number of operating slip systems and an increased level of DRV as the temperature increased [4]. Figure 6b reveals that the density of low angle boundaries was significantly reduced, and subgrains with higher angle boundaries (5°–15°) were found along the grain boundaries, thus indicating a further recovered structure. In Domain III, the grain boundaries of deformed samples remained planar and deformation bands were not noticeable, as an example shown by the sample deformed at 350 °C and 0.001 s^−1^. Moreover, a strong recovered microstructure was observed inside the elongated grains (Figure 6c). The EBSD result shows that the substructure became better organized, and a number of larger subgrains were formed with neatly arranged boundaries, which were characterized by misorientation angles between five and 15° (Figure 6d). This suggests an increased level of DRV as the strain rate decreased, involving the annihilation and rearrangement of dislocations.

**Figure 7.**
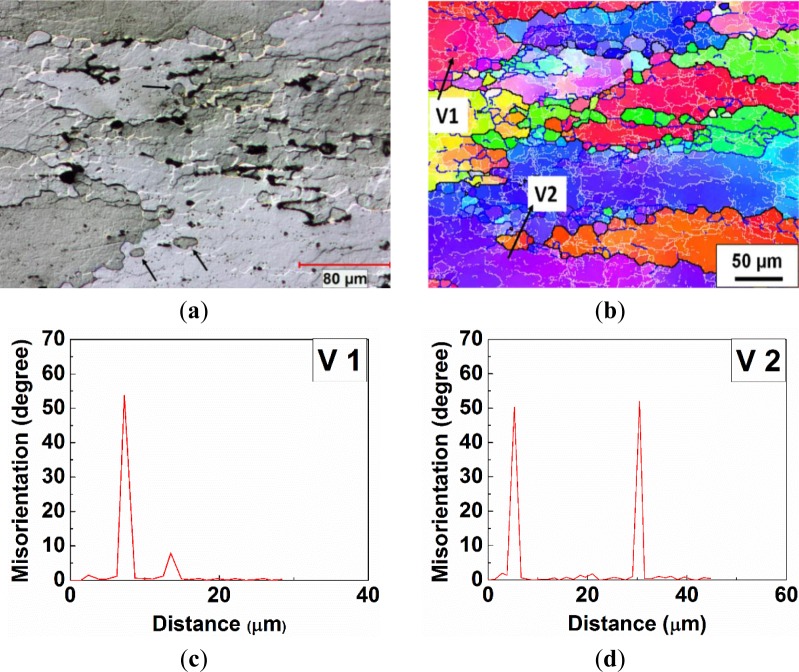
Deformed microstructures under deformation condition at 450 °C, 0.01 s^−1^: (**a**) optical micrograph; (**b**) orientation imaging map; (**c**) a misorientation profile along vector V1; and (**d**) a misorientation profile along vector V2.

In Domain IV, during the deformation at 450 °C and 0.01 s^−1^, the bulging of original grain boundaries was frequently observed, and small equiaxed grains were found along the serrated grain boundaries (see the arrows in Figure 7a), indicating a partially recrystallized microstructure. An example is given by a misorientation profile along Domain V1 (Figure 7b,c), where a bridging medium angle boundary (8°) has been developed behind a bulged section of the boundary of an original grain (54°). Furthermore, as the deformation proceeded, a bulged section was completely pinched off and became a refined grain, which contained a substructure and possessed a similar orientation to its parent grain (V2 in Figure 7b,d). The average size of the recrystallized grains is approximately 14 μm. It is approximately two times the size of the subgrains (5–7 μm) reported in 7050 and 7075 aluminum alloys at the same deformation condition, in which DRV solely operated as the softening mechanism [22,46]. This result is in agreement with the study of 5083 aluminum alloy during hot torsion by McQueen *et al.* [44], in which the size of the dynamic recrystallized grain is 2–3 times the subgrain size. Therefore, partial DRX occurred during the hot deformation, and little grain rotation was involved in the formation of those recrystallized grains.

**Figure 8.**
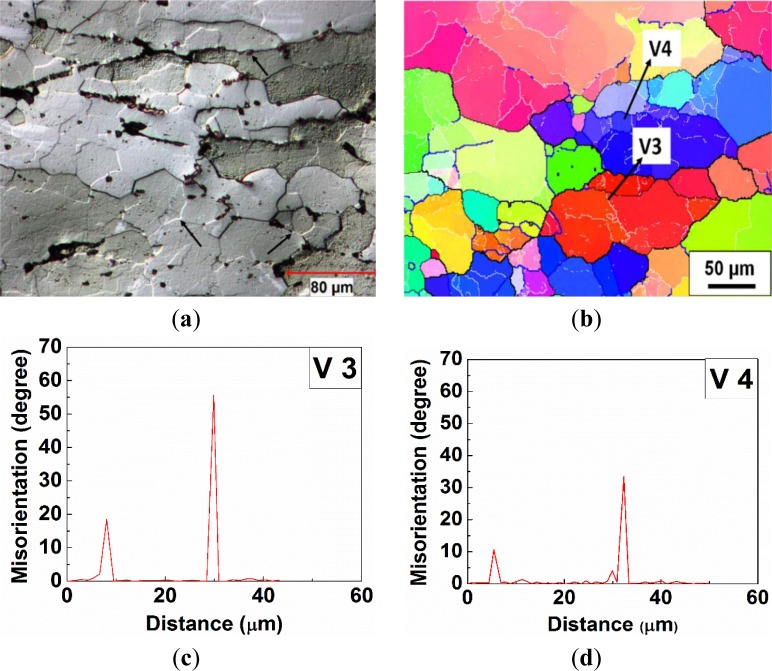
Deformed microstructures under deformation condition at 450 °C, 0.001 s^−1^: (**a**) optical micrograph; (**b**) orientation imaging map; (**c**) a misorientation profile along vector V3; and (**d**) a misorientation profile along vector V4.

In Domain V, when the deformation was performed at 450 °C and 0.001 s^−1^, new grains with high angle boundaries containing substructures were presented along the elongated original grain boundaries (see the arrows in Figure 8a), and a large number of subgrains were observed inside the original grains, as well, which illustrated a strong dynamic recovered microstructure mixed with a dynamic recrystallized microstructure (Figure 8a,b). The microstructure of the recrystallized grain consists of partially the original grain boundaries (30°–60°) and the newly formed high angle boundaries (15°–30°), as illustrated by V3 in Figure 8b,c. Besides, a misorientation profile, V4 in Figure 8b,d, demonstrates that a subgrain was progressively increasing in misorientation and being transformed into a dynamically recrystallized grain. Moreover, those recrystallized grains exhibited an average size of 33 μm, which is about three times the subgrain size (9.5 μm) solely observed in 7050 aluminum alloy during deformation at 450 °C and 0.0005 s^−1^ by Deng *et al.* [46].

### 3.5. Quantitative Analyses of Grain Boundaries

**Figure 9.**
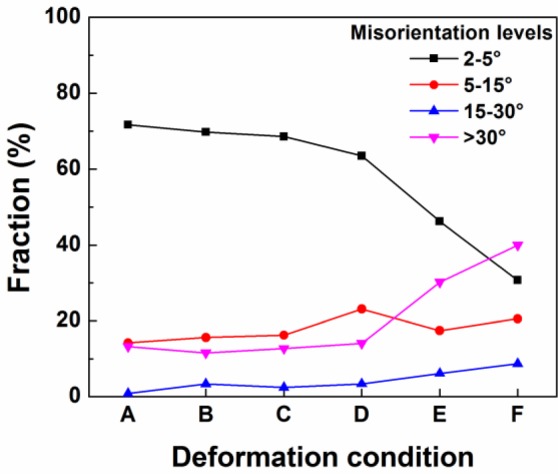
Fractions of boundaries with different misorientation levels under different deformation conditions: A, 300 °C, 10 s^−1^; B, 300°C, 1 s^−1^; C, 350 °C, 0.1 s^−1^; D, 400 °C, 0.01 s^−1^; E, 450 °C, 0.01 s^−1^; and F, 450 °C, 0.001 s^−1^.

Based on the microstructure observation of samples deformed at various deformation conditions, DRV generally occurred during hot deformation in Domains I, II and III, and DRX took place when the deformation was conducted in Domains IV and V. For a further investigation of the dynamic softening mechanisms during the hot deformation processes, a quantitative measurement of misorientation angle distributions of boundaries for samples deformed at different deformation conditions was carried out using the EBSD technique, and the results are illustrated in Figure 9. It is evident that at deformation temperatures between 300 and 400 °C, a continuous decrease in the fraction of low angle boundaries (1°–5°), along with an increase in the fraction of medium angle boundaries (5°–15°) was observed as the temperature increased and the strain rate decreased (see the deformation conditions from A to D). This implies an increased level of DRV, involving the elimination of low angle boundaries and transformation into higher angle boundaries, which is consistent with the microstructural evolution observed in Figures 5 and 6. However, there is no indication of DRX occurring during the deformation at temperatures of 300 to 400 °C, due to the little variation in the fraction of high angle boundaries (>15°). On the other hand, as the temperature increased to 450 °C with different strain rates of 0.01 s^−1^ and 0.001 s^−1^ (deformation conditions E and F), the boundaries with misorientation angles between one and 15° decreased dramatically and were associated with a rapid increase in high angle boundaries. This result suggests that DRX occurred accompanied with an increasing fraction of high angle boundaries, as a result of the formation of recrystallized grains. This is in good agreement with the microstructure observation in Figures 7 and 8.

## 4. Discussion

The decline ratio map of flow stress as a function of temperature and strain rate is divided into five domains, which can be utilized to study the relationship between flow stress behavior and various dynamic softening mechanisms in the Al-Zn-Mg-Cu alloy (7150) during hot deformation at different deformation temperatures and strain rates.

**Figure 10.**
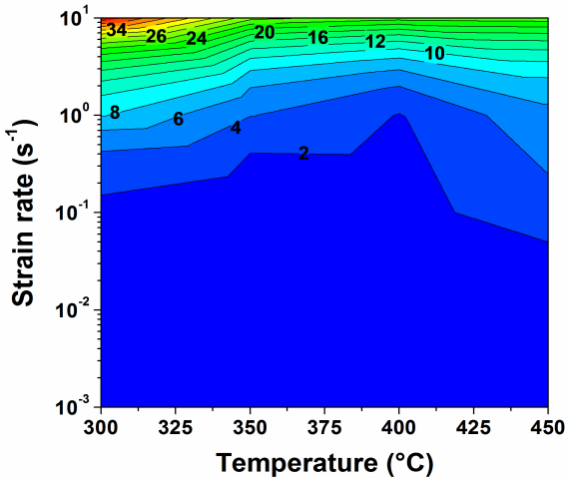
Maximum increase of sample temperature (Δ*T*_max_) measured during compression tests as a function of deformation temperature and strain rate.

In Domain I, the microstructure observation confirms that DRV is the main dynamic softening mechanism during hot deformation (Figure 5). Although deformed at high strain rates, no cracking was observed in the deformed samples. The continuous decline of the flow stress after attaining the peak stress is likely associated with a severe deformation heat release. The variations of sample temperatures are shown in Figure 10, which were monitored during the compression tests at the target temperatures from 300 to 450 °C with different strain rates. When the deformation was performed at the high strain rate of 10 s^−1^ (Domain I), the sample temperature rose continuously with increasing strain at each target deformation temperature. The maximum overheating temperature reached 22 °C at the target temperature of 450 °C and increased as high as 35 °C at the target temperature of 300 °C. Those results suggest that the heat release during deformation at the high strain rate of 10 s^−1^ plays an important role in the decrease of flow stress, leading to a significant thermal softening, as confirmed by other researchers [16–18].

However, in other domains, when the deformation was carried out at low strain rates between 0.001 s^−1^ and 0.1 s^−1^, the sample temperatures kept close to the target temperatures (within 2 °C). Thus, it is reasonable to consider that the deformation was under an isothermal process. Even at the intermediate strain rate of 1 s^−1^, the temperature increase was generally limited within several degrees (4 to 8 °C). Therefore, it is believed that the thermal softening effect in this case plays a minor role in the flow softening.

Domain II represents a typical recovered structure (Figure 6a,b), indicating that DRV is the sole dynamic softening mechanism. The flow stress curves showed a continuous increase of flow stress after the peak stress at a higher strain rate (1 s^−1^) or a slight decrease at a lower strain rate (0.1 s^−1^). It is evident that the effect of work hardening was dominant during hot deformation at the strain rate of 1 s^−1^, whereas the dynamic softening slightly overcame the work hardening at the lower strain rate of 0.1 s^−1^.

**Figure 11.**
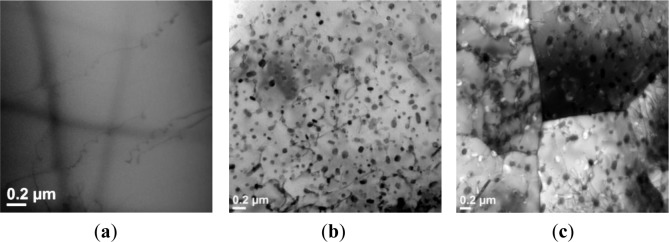
Bright-field TEM micrographs showing: (**a**) as-homogenized microstructure prior to deformation; and deformed microstructures at 350 °C and 0.001 s^−1^ with the true strain of (**b**) 0.1, and (**c**) 0.8. The electron beam is parallel to [011]_α_.

In Domain III, DRV still operated as the main dynamic softening mechanism (Figure 6c,d). Differentiated from Domain II, a significant decline of the flow stress after the peak stress occurred. It was observed that the dynamic precipitation and coarsening took place during the hot compression process. Figure 11a shows a precipitate-free aluminum matrix after the homogenization prior to the hot deformation. At the beginning of deformation (at a true strain of 0.1), as exemplified at the deformation condition of 350 °C and 0.001s^−1^, a large number of spherical and rod-shaped precipitates with an average size of 60 nm appeared (Figure 11b). The TEM-EDS result (Table 2) shows that the precipitates contain Mg, Zn and Cu, with a composition approaching the stoichiometric Mg(Zn,Cu)_2_ phase [47–49]. Those fine precipitates interacted with dislocations and exhibited a strong pinning effect on dislocation movement, leading to a high value of the peak stress at the initial stage of deformation. As the deformation progressed to a true strain of 0.8, the precipitates remarkably coarsened to an average size of 125 nm with a larger interparticle spacing (Figure 11c). The coalescence of precipitates, accompanied by enhanced DRV, resulted in a significant flow stress softening. Similar behaviors have been observed in solution treated 7012, 7075 and 7085, as well as homogenized 2026 aluminum alloys during hot deformation at low strain rates [17,22,24].

It should be mentioned that dynamic precipitation can also occur in Domain II. However, the coalescence of precipitates was not observed by TEM, due to a relatively short deformation time at high strain rates. Those fine precipitates significantly increased the multiplication rate of dislocations [2,4], which resulted in a strong work hardening effect and a continuous increase of flow stress at a strain rate of 1 s^−1^, as shown in Figure 2.

**Figure 12.**
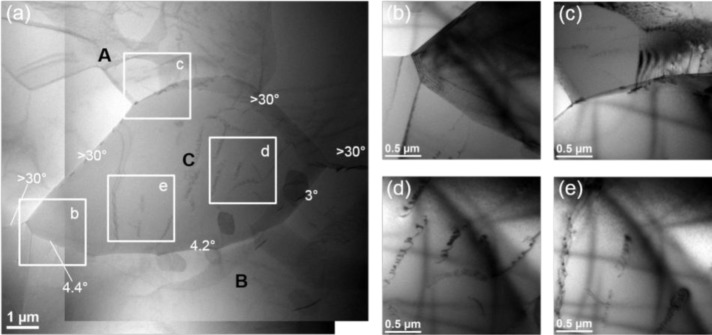
Transmission electron micrographs of the alloy compressed at 450 °C and 0.01 s^−1^ to a true strain of 0.8: (**a**) STEM image showing one dynamically recrystallized grain and its surroundings; and (**b**–**e**) bright-field TEM images illustrating the enlarged views of four locations in (**a**).

Moreover, in Domain IV, the deformed microstructure reveals a partially recrystallized microstructure at the serrated grain boundaries (Figure 7), which probably involves the discontinuous dynamic recrystallization (DDRX) by strain-induced boundary migration (SIBM). Figure 12 illustrates an example of the formation of one dynamically recrystallized grain before pinching off from the original grain in the sample deformed at 450 °C and 0.01 s^−1^. The scanning transmission electron microscope (STEM) micrograph gives an overview of the recrystallized grain and its surroundings, and four bright-field TEM images show the enlarged views in the corresponding areas. The misorientation angles of the boundary were determined by measuring the shift of the Kikuchi line intersections on the crossing boundary under the TEM mode [2,23].

It can be seen that the recrystallized grain C is comprised of a partially bulged original grain boundary (Figure 12a,c) and a bridging of the low angle boundary (3°–5°) with grain B (Figure 12a,b). This suggests a characteristic feature of SIBM, by which the grain C was gradually formed at the expense of the higher density of dislocations in grain A and displayed a similar orientation to its parent grain, B. Moreover, grain C contains substructures of dislocations and dislocation networks (Figure 12a,d,e). This provides direct evidence that the new grain was formed dynamically during deformation and suffered a significant strain [2,7]. Furthermore, it was reported that a high content of solute atoms, Mg, Zn and Cu in the aluminum matrix of the homogenized sample, could reduce the dislocation mobility and retard dynamic recovery, thus leading to a high stored energy [2,4,12]. Therefore, the driving force for dynamic recrystallization was increased and DDRX took place during the hot deformation process at this deformation condition, resulting in an appreciable flow softening [2,12].

**Figure 13.**
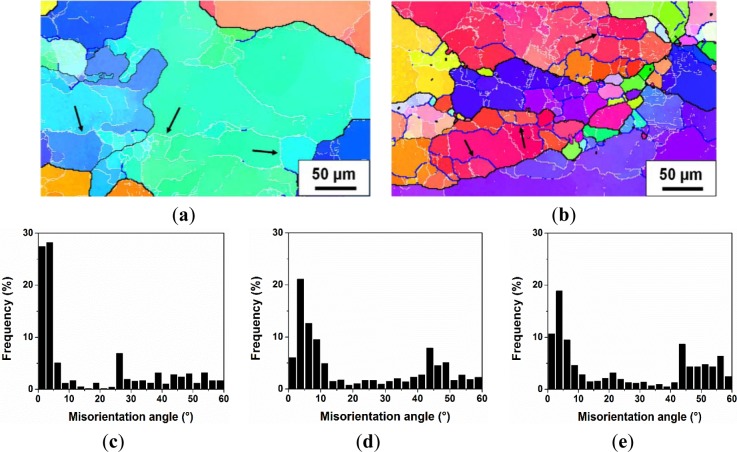
Orientation imaging maps of the alloy deformed at 450 °C and 0.001s^−1^ with the true strain of: (**a**) 0.3 and (**b**) 0.5; misorientation distribution at the true strain of: (**c**) 0.3, (**d**) 0.5 and (**e**) 0.8.

During the deformation at a high temperature of 450 °C and at a low strain rate of 0.001 s^−1^ in Domain V, the bulging of grain boundaries and the formation of recrystallized grains by DDRX were not observed in Figure 8. As a high level of DRV was induced during the slow straining process at the high temperature, the accumulation rate of the dislocations was considerably decreased. Hence, the driving force was not sufficient for nucleation and the growth of recrystallized grains by DDRX. However, a number of newly formed grains with high angle boundaries can be clearly seen in the microstructure (Figure 8), in which it most probably involved a continuous dynamic recrystallization (CDRX). Figure 13 shows the evolution of the substructure and misorientation distribution with increasing strain during the deformation at 450 °C and 0.001 s^−1^. At a true strain of 0.3, subgrains were well developed inside the original grains (see the arrows in Figure 13a) and characterized mainly by low angle boundaries between one and 5° (Figure 13c). As the deformation was processed to a true strain of 0.5, subgrains were presented with boundaries of increased misorientations (see the arrows in Figure 13b). Besides, the fraction of subgrain boundaries with low misorientations (1°–5°) was significantly reduced, while the number of boundaries with higher misorientations increased (Figure 13d). When the true strain approached 0.8, recrystallized grains with high angle boundaries could be observed (Figure 8), and the fraction of high angle boundaries continuously increased, reaching more than 45% (Figure 13e). This result indicates that the low angle boundaries (1°–5°) were progressively converted into medium angle boundaries (5°–15°), which, in turn, transformed into high angle boundaries during the slow deformation process (Domain V). Such an evolution can be considered as a process of continuous dynamic recrystallization (CDRX) [10–12]. In this case, the flow stress curve attained a peak stress and then showed a slow decline upon further straining [50].

In brief, the decline ratio map of flow stress was utilized to study the relationship between flow stress behavior and various dynamic softening mechanisms in the Al-Zn-Mg-Cu alloy (7150) during hot deformation. Recovered structures are generally observed in the samples deformed in Domains I, II and III. In these cases, DRV is the sole dynamic softening mechanism during hot deformation, which is commonly reported in 7xxx aluminum alloys [2,22,46,51]. However, a partially recrystallized microstructure is presented in the deformed samples in Domains IV and V. It is proved using EBSD and TEM that DRX does take place during hot deformation at certain conditions in this alloy. Therefore, besides DRV, DRX plays an important role in flow stress softening at the high temperature. Furthermore, results reveal that two possible mechanisms of DRX operate during hot forming, in which DDRX is involved in Domain IV and CDRX is implied in Domain V. Finally, it should be emphasized that the generation of a decline ratio map of flow stress, accompanied by investigation on typical microstructures in each domain, could provide a convenient approach to study the dynamic softening mechanisms of materials through hot processing.

Recently, several works have been done to optimize the hot working processes of 7xxx aluminum alloys using the processing maps [52–55]. Correlating various deformation mechanisms suggested from the decline ratio map with those processing maps, it is possible to predict the hot workability of the Al-Zn-Mg-Cu aluminum alloy studied. The results obtained in this study show that Domains II, IV and V correspond to the safe processing domains, due to DRV and DRX operating as the dynamic softening mechanisms [42,52–55]. Furthermore, the optimum hot working parameters for the alloy are considered to be in Domains IV and V, i.e., at the temperature of 450 °C and strain rates of 0.001–0.1 s^−1^, where DRX occurs during hot deformation, leading to softening and the reconstituting of the microstructure [42]. This is in agreement with the results obtained from 7xxx aluminum alloys by other researchers [52–55]. On the other hand, the deformation in Domain I should be avoided, due to the flow instability and the formation of adiabatic shear bands [42,54,55]. The coarse dynamic precipitates generated in Domain III may lead to the deep inter-granular corrosion and large areas of the denudation layer, which are detrimental to the properties of the deformed material [54].

## 5. Conclusions

1. The hot deformation behavior of an Al-Zn-Mg-Cu alloy (7150) is systematically investigated at temperatures of 300 to 450 °C and strain rates of 0.001 to 10 s^−1^. The level of flow stress decreases with an increasing deformation temperature and with a decreasing strain rate.
2. The decline ratio map of flow stresses is proposed and further divided into five deformation domains, in which the flow stress behavior is correlated with different microstructures and dynamic softening mechanisms at various deformation conditions.
3. The dynamic recovery is the sole softening mechanism of the alloy when the hot deformation is performed at temperatures of 300 to 400 °C with various strain rates, as well as at temperatures of 400 to 450°C with strain rates between one and 10 s^−1^. The level of dynamic recovery increases with increasing temperature and with decreasing strain rate.
4. At the high strain rate of 10 s^−1^, the heat release during deformation plays an important role in the decrease of flow stress, leading to a significant thermal softening. At low strain rates of 0.001 to 0.01 s^−1^ and temperatures of 300 to 400 °C, the coalescence of dynamic precipitation, accompanied by enhanced dynamic recovery, results in a considerable decline in flow stress.
5. The dynamic recrystallization takes place when the deformation is conducted at the high deformation temperature of 450 °C with strain rates of 0.001 to 0.1 s^−1^, providing an alternative softening mechanism. The results reveal that two kinds of DRX may operate at the high temperature, in which DDRX is involved at the higher strain rates and CDRX is implied at the lower strain rates.

## Figures and Tables

**Table 1.** Chemical compositions of the experimental alloy (wt%).

Material	Chemical compositions (wt%)								
	Zn	Mg	Cu	Si	Fe	Mn	V	Ti	Al
Al-Zn-Mg-Cu Alloy	6.44	2.47	2.29	0.16	0.15	0.002	0.01	0.009	Balance

**Table 2.** Results of TEM-energy dispersive spectroscope (EDS) analysis of the precipitates shown in Figure 11b,c.

Phase	Chemical compositions (mole fraction, %)			
	Mg	Cu	Zn	Al
Mg(Zn,Cu)_2_	26.58	18.82	25.67	28.94
